# Adjunctive benefits of low-frequency transcutaneous electrical nerve stimulation for obesity frequent chronic conditions: a systematic review

**DOI:** 10.3389/fendo.2024.1424771

**Published:** 2024-08-09

**Authors:** An Yu, Xiang Li, Wei Zhang, Yazhou Zhang, Xi Chen, Liuyan Wang, Mei Xie, Lei Yang

**Affiliations:** ^1^ Yunnan Key Laboratory for Basic Research On Bone and Joint Diseases &, Yunnan Stem Cell Translational Research Center, Kunming University, Kunming, Yunnan, China; ^2^ Department of Rehabilitation Medicine, The Second People’s Hospital of Kunming, Kunming, Yunnan, China; ^3^ Department of Rehabilitation Medicine, Yan An Hospital of Kunming City, Kunming, Yunnan, China; ^4^ Department of Geriatrics, The Second People’s Hospital of Kunming, Kunming, Yunnan, China

**Keywords:** obesity, essential hypertension, type 2 diabetes mellitus, dyslipidemia, transcutaneous electrical nerve stimulation, systematic review

## Abstract

**Background:**

Obesity is widely recognized for its role in predisposing individuals to a spectrum of chronic health conditions. Emerging preliminary evidence points to the potential benefits of low-frequency transcutaneous electrical nerve stimulation (Lo-TENS) in enhancing various health outcomes among those with obesity and associated disorders.

**Objective:**

This systematic review was designed to assess the effectiveness of Lo-TENS for managing obesity and its related chronic diseases.

**Methods:**

For this systematic review, we included randomized controlled trials that evaluated the impact of Lo-TENS on individuals with obesity and its associated chronic diseases.

**Results:**

Eight trials encompassing 671 participants and spanning three unique populations: essential hypertension (EH), type 2 diabetes mellitus (T2DM), and obesity were deemed eligible for inclusion in this review. Compared to baseline measurements, Lo-TENS demonstrated a tendency to positively affect blood pressure in individuals with EH and metabolic parameters in those with T2DM. Nonetheless, the efficacy of Lo-TENS in treating obesity is not yet clear when contrasted with a no-intervention control group. When compared with other intervention modalities, three of the trials reported less favorable results.

**Conclusions:**

Although Lo-TENS did not consistently surpass other treatments or yield substantial improvements, it generally provided greater benefits than the majority of placebo controls. This suggests that Lo-TENS could potentially serve as a beneficial adjunctive therapy in the management of obesity and its associated conditions. However, given the limited number of trials assessed, the elevated risk of bias within these studies, and the scarce evidence currently available, it is too early to reach definitive conclusions. Caution should be exercised when interpreting the current findings. There is an imperative for further high-quality research to thoroughly investigate and substantiate the efficacy of Lo-TENS in relation to obesity and its related disorders.

## Introduction

Over the past few decades, obesity has seen a marked rise in both prevalence and severity, becoming a global health issue. It is widely acknowledged that obesity is not only associated with a diminished quality of life and heightened risk of early mortality but also predisposes individuals to an array of chronic conditions. These include, but are not limited to, essential hypertension (EH), cardiovascular diseases, type 2 diabetes mellitus (T2DM), dyslipidemia, hyperglycemia, and certain types of cancer (1). The substantial medical and societal expenses stemming from the complications of obesity continue to pose significant challenges to healthcare systems worldwide (2).

Lifestyle interventions, including weight reduction and increased physical activity, are established as the primary approach to managing obesity (3, 4). However, achieving and maintaining weight loss is frequently challenging (5). In light of the difficulties associated with long-term weight management, it is crucial to explore and implement innovative strategies that enhance weight loss efforts and curb the advancement of obesity-related chronic conditions.

Obesity is a multifaceted condition with numerous contributing factors. Research has increasingly highlighted the link between obesity and the persistent activation of the sympathetic nervous system (6, 7), which plays a pivotal role in its metabolic ramifications. Evidence suggests that the activation of the sympathetic nervous system is a key early event in the progression of obesity and is implicated in the emergence of metabolic disorders, including hypertension and endothelial dysfunction (8, 9). Furthermore, studies have demonstrated a correlation between heightened sympathetic activity and the onset of hypertension, hyperglycemia, hyperlipidemia, and metabolic syndrome (10, 11). Prolonged activation of the sympathetic nervous system can precipitate detrimental metabolic consequences, such as the rapid release of glucose from the liver and the reduction of insulin levels in the portal blood (12), coupled with an increase in glucagon concentration (13). There is growing recognition that obesity may disrupt the sympathetic regulation of cardiovascular function, potentially leading to an increased risk of cardiovascular complications and events (10, 11).

Given the complex pathophysiology outlined earlier, it has been suggested that strategies aimed at inhibiting sympathetic activation could potentially lead to weight loss and mitigate the risks associated with chronic diseases (2). Traditional methods for achieving this inhibition encompass pharmacological interventions and device-based treatments (14, 15). However, since comorbidity is common in people with obesity, poly-pharmacy is not rare in these people, leading to the side effect and poly-pharmacy interaction (14). Consequently, there is a pressing need to explore alternative and complementary therapies that can support weight reduction and manage chronic conditions, ultimately enhancing the overall quality of life for these individuals.

Over the past decade, there has been a surge of interest in device-based therapies aimed at inhibiting the activity of the sympathetic nervous system (16). Transcutaneous electrical nerve stimulation (TENS) is a technique that employs electrical stimulation at an intensity below the threshold for motor response. In general, TENS is categorized into high-frequency TENS (Hi-TENS, 50~150 Hz) and low-frequency TENS (Lo-TENS, 1~20 Hz) (17, 18). Traditionally, Hi-TENS is usually used for pain relief by blocking afferent nerve signals. However, research has revealed that TENS, when applied at lower frequencies (2-4 Hz), may also serve to reduce sympathetic nerve activity (17). Comparative study has shown that Lo-TENS, when applied to the area of the paraventricular ganglion in hypertensive patients, can effectively reduce sympathetic nervous system activity and boost parasympathetic nervous system activity, leading to a decrease in diastolic blood pressure. Conversely, Hi-TENS has been observed to increase diastolic blood pressure. This study indicates that Lo-TENS can decrease sympathetic nerve activity and enhance parasympathetic nerve activity, whereas Hi-TENS tends to elevate sympathetic nerve activity. These findings underscore the potential of Lo-TENS as a therapeutic tool for modulating autonomic balance beyond its established analgesic applications (18).

Hence, it is believed that Lo-TENS may serve as an adjunctive treatment for obesity and associated chronic diseases by countering the excessive activity of the sympathetic nervous system. To the best of our knowledge, no systematic review or meta-analysis has yet explored the impact of Lo-TENS on these conditions. This review is designed to fill that void by examining the available evidence regarding the efficacy of Lo-TENS in managing obesity and associated chronic diseases, thereby contributing to the understanding of its potential role as a supplementary therapy.

## Methods

### Search strategy

This systematic review was conducted following the PRISMA (Preferred Reporting Items for Systematic Reviews and Meta-Analyses) guidelines (19). Two researchers, (A.Y. and X.L.) independently conducted a comprehensive electronic literature search across various databases, including PubMed, Medline, Cochrane Library, Web of Science, and CINAHL. The search utilized a combination of keywords related to essential hypertension, diabetes mellitus, dyslipidemia, hypercholesterolemia, obesity, metabolic syndrome, and transcutaneous electrical nerve stimulation. The specific search strategy for PubMed is detailed in **Appendix 1**, and analogous approaches were applied to the other databases.

### Inclusion and exclusion criteria

In alignment with the PICOS framework, the following inclusion criteria were established for this review (1): Participants: adults diagnosed with prevalent chronic conditions such as EH, T2DM, dyslipidemia, obesity, and metabolic syndrome; (2) Intervention: the treatment group received Lo-TENS, with the intervention protocol described in detail, e.g., frequency, stimulation sites, intensity, duration, etc.; (3) Comparison: the control group either received no intervention (placebo-controlled) or an alternative intervention (e.g., different TENS parameters, pharmacological treatments, physical therapy, etc.); (4) Outcomes: the primary outcomes of interest were blood pressure, glycemic levels, lipid profiles, and body mass index (BMI). Secondary outcomes included any metrics pertinent to the management and effects of hypertension, diabetes, dyslipidemia, obesity, and metabolic syndrome; (5) Study design: randomized controlled trials (RCTs).

Exclusion criteria were as follows: (1) studies for which the full text could not be accessed, despite attempts to contact the original author; (2) non-English articles; (3) the articles used the duplicated data.

### Article selection

Two reviewers (A.Y. and X.L.) independently assessed the relevance of the articles by examining their titles and abstracts. Eligibility was then confirmed by a full-text review. Any discrepancies between the reviewers were resolved through consultation with the principal investigator (L.Y). The references of the selected articles were scrutinized for additional relevant studies. Furthermore, a forward citation search was performed using the Web of Science to ensure the comprehensive inclusion of all pertinent articles. The final updated search was completed on March, 2024.

### Assessment of methodological quality

The methodological quality of the included trials was evaluated using the Cochrane Risk-of-Bias Tool (version 2.0), which assesses the potential for bias across various domains (20). Trials were categorized as having a low, high, or some concerns for risk of bias in each domain. A trial was considered to have a low risk of bias if all assessed domains were rated as low risk. Conversely, a high risk of bias was assigned if there was a high risk in at least one domain or multiple domains had some concerns. Two independent investigators (A.Y. and X.L.) collaboratively assessed and rated the risk of bias for each trial, any disagreement between them was discussed and resolved with the principal investigator (L.Y).

### Data extraction and synthesis

After reading the full text, data extraction and synthesis were conducted in terms of participant characteristics, intervention protocols, outcome measurements, etc. In order to better summarize the different kinds of outcomes, measures were synthesized according to different populations and classified into anthropometric, blood pressure, lipid, glycemia, or exploratory domain.

## Results

### Article selection and methodology assessment

**Figure 1:**
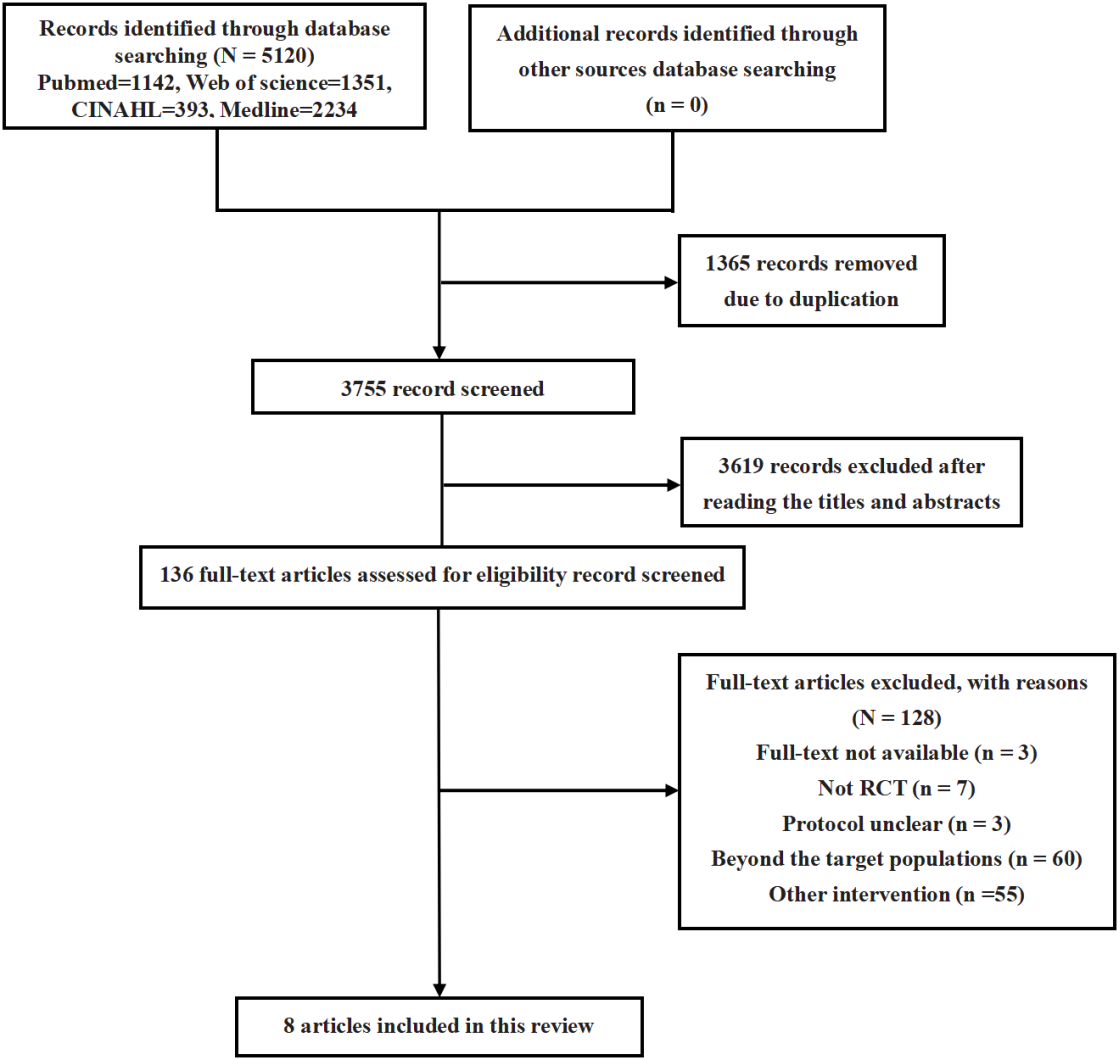
Flow Chart of trials screening.

**Figure 2:**
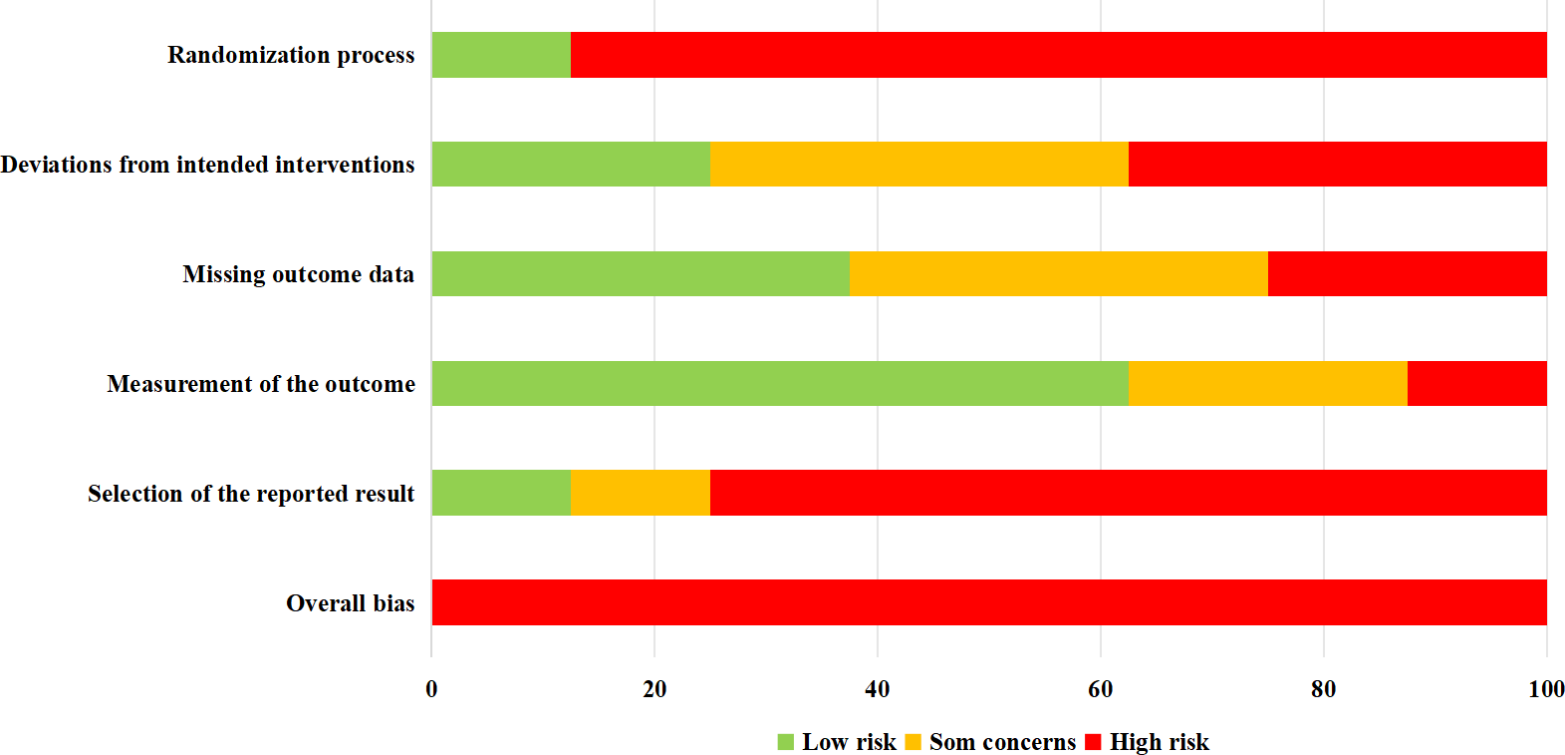
Risk of bias of studies using Cochrane Risk of Bias Tool (V2).

Following an extensive search of electronic databases and subsequent screening of articles, this review included eight studies (comprising eight trials) (18, 21–27). The process of article selection is depicted in **Figure 1**. An assessment of the methodological quality of the included studies is presented in **Figure 2**. It was found that all trials exhibited a high risk of bias (18, 21–27).

### Participants’ characteristics and intervention protocols

The study population comprised a total of 671 participants across three distinct populations: mild to moderate EH (four trials, N=166) (18, 21–23), T2DM (two trials, N=245) (24, 25), and obesity (two trials, N=260) (26, 27). The average age of participants ranged from 38.5 (10.6) to 63.9 (9.2) years. Further details regarding the demographic and clinical characteristics of the study participants can be found in **Table 1**.

**Table 1:** Characteristics of participants and intervention protocols (N=8).

Study	Characteristics of Participants		Intervention Protocols			
	Sample Size (ratio of female) [Age (y), Mean (SD)]	Key inclusion criteria	Experimental group			Comparison group
			Channel Electrodes placement	TENS parameters	Intervention Volume	
Kaada et al. (21), 1991	46 (34.8%) Intervention group: n=25 (41~43); Control group: n=21 (41~43)	DBP: 90~115	Single channel One on the dorsal web between the first and second metacarpal bones, the other on the ulnar border of the same hand	**Wave form:** biphasic square wave **Frequency:** 2 Hz **Pulse width:** 0.2 ms **Intensity:** local, rhythmic contractions of the fingers, without pain (usually at 15-30 mA)	**Short-term study:** 45 min, single session **Long-term study:** 45 min × 2 times/d × 2 weeks	Sham control: the electrodes were connected, but no current was delivered
Tu et al. (23), 2021	60 (60%) TENS group: n=30 [58.5 (52.0, 61.0)] Control group: n=30 [60.0 (57.0, 63.0)]	Grade 1 hypertension	Dual channel In the first session, one channel to the ipsilateral LI4 (Hegu) and LI11 (Quchi) acupoint of the forearm, while the other channel to the contralateral side; in the second session, one channel to the ST36 (Zusanli) and LR3 (Taichong) acupoints of the leg, while the other channel to the contralateral side.	Not reported	15 min × 2 times/d × 4 times/week × 2 weeks	Usual care: maintaining participants’ medication and performing health education
Silverdal et al. (22), 2012	32 (25%) (crossover design) (55)	SBP: 140~170 DBP: 90~105	Dual channel One channel to the ipsilateral dorsal web between the first and second metacarpal bones, and on two finger breadths distal to the radial part of the bent arm (i.e., corresponding to acupuncture points of LI 4 and LI 10), while the other channel to the contralateral side.	**Wave form:** biphasic asymmetrical wave **Frequency:** 2 Hz **Pulse width:** not reported **Intensity:** individually adjusted, to trigger contractions of muscles without reaching painful levels	30 min × 2 times/d × 4 weeks	Felodipin: 2.5 mg/d x 4 weeks
Sartori et al. (18), 2018	28 (67.9%) Lo-TENS: n=8 [58.1 (8.4)] Hi-TENS: n=10 [59.4 (10.0)] Control group: n=10 [59.8 (10.7)]	SBP: > 140 and/or DBP: > 90	Dual channel In the bilateral paravertebral ganglionar region from T1 to L2	**Wave form:** not reported **Frequency:** low (4 Hz), high (100 Hz) **Pulse width:** 200 μs **Intensity:** at sensory-level, but without motor contraction or pain reported	30 min, single session	Hi-TENS: using 100 Hz current to stimulate, the other parameters were the same; Control group: placebo treatment.
Lu et al. (25), 2023	155 (42.6%) TENS group: n=78 [60 (10)], Placebo group: n=77 [59 (9)]	Type 2 diabetes	Single channel Bilateral abdominal wall	**Wave form:** monophasic square pulse wave with 50% duty cycle **Frequency:** full-frequency wave resonant with mixed frequencies ranging from 1 to 20,000 Hz **Pulse width:** not reported **Intensity:** 7.2 Vpp in average	60 min × 5 times/week × 20 weeks	Control group: full-frequency wave resonant with mixed frequencies ranging from 1 to 30 Hz.
Wu et al. (24), 2015	90 (61.1%) Acu-TENS group: n=30 [63.9 (9.2)], Control group: n=30 [63.2 (10.4)], Aerobic exercise group: n=30 [63.5 (9.4)]	Type 2 diabetes	Four channels Pairs of electrodes were separately fixed at bilateral Quchi (LI 11), Hegu (LI 4), Zusanli (ST 36), and Sanyinjiao (SP 6).	**Wave form:** discontinuous wave (muscle contraction 7 times with an interval of 8 seconds between contractions) **Frequency:** 10 Hz **Pulse width:** not reported **Intensity:** 5 mA	30 minutes × 5 times/week × 2 months	Sham control group: The control group treatment was no different from the Acu-TENS group except that no current flowed through the electrodes. Aerobic exercise group: received walking training. Training lasted for 30 min, five times a week, for 2 months.
Choi et al. (27), 2018	60 (76.7%) TENS group: n=30 [40.0 (12.9)], Electrical muscle simulation group: n=30 [38.5 (10.6)],	Overweight	Channels: not reported. Electrodes were applied to the rectus abdominis and external oblique abdominal muscle areas.	**Wave form:** not reported **Frequency:** 1 Hz **Pulse width:** 150 μs **Intensity:** caused muscle movement, but not an effective muscle contraction exercise	66 min × 5 times/week 12 weeks	Electrical muscle simulation group: warm-up (5 Hz, 250 μs, action 2 sec, resting 3 sec, 3mins/cycle × 2 times, total 6 min), contraction (55 Hz, 300 μs, action 10 sec, resting 10 sec, 10 min/cycle × 2 times, total 20 min), warm-up (6 Hz, 180 μs, action 2 sec, resting 3 sec, 5 min/cycle × 2 times, total 10 min), contraction (65 Hz, 300 μs, action 10 sec, resting 10 sec, 10 min/cycle × 2 times, total 20 min), warm-down (4 Hz, 160 μs, action 2 sec, resting 3 sec, 5 min/cycle × 2 times, total 10 min)
Ruiz-Tovar et al. (26), 2017	200 (80%) TENS+hypocaloric diet group: n=50 [45.1 (10.9)], PENS+normocaloric diet group: n=50 [44.9 (9.9)], PENS+normocaloric diet group: n=50 [45.2 (10.8)], Hypodiet Group: n=50 [45.3 (10.4)]	Obesity	Channels: not reported. Electrodes were placed in the right iliac fossa.	**Wave form:** not reported **Frequency:** 20 Hz **Pulse width:** not reported **Intensity:** at the highest amplify (0 to 20 mA) without causing pain.	30 minutes × 1 times/week × 12 weeks	PENS group: The needle electrodes were inserted in the left upper quadrant along the medioclavicular line, 2 cm below the rib cage. PENS was undertaken at frequency of 20 Hz at the highest amplify (0 to 20 mA) without causing pain. Diets: Diets prescribed [hypocaloric (1200 kcal/d) and normocaloric (2000 kcal/d)] were based on Mediterranean diet patterns. Dietary compliance was evaluated by means of a food diary that the patients filled. Diet and neurostimulation started at the same time.

N, number of included trials; NR, not reported.

Acu-TENS, acupoint transcutaneous electrical nerve stimulation; DBP, diastolic blood pressure; Hi-TENS, high frequency transcutaneous electrical nerve stimulation; Lo-TENS, low frequency transcutaneous electrical nerve stimulation; SBP, systolic blood pressure.

### Intervention protocols

The intervention protocols showcased variability among the different trials and participant groups. A comprehensive overview of the intervention parameters is provided in **Table 1**. Among the trials, two did not specify the number of channels used (26, 27). The remaining trials employed one (21, 25), two (18, 22, 23), or four channels (24). One particular trial focused on stimulating the paravertebral ganglionar region (from T1 to L2) (18), while the rest targeted peripheral sites such as forearms, abdomen, or acu-points. With the exception of Tu et al. (23), the other trials documented their stimulation parameters, including waveform, frequency, and intensity. However, there was a lack of uniformity in these parameters across the trials. Two trials were designed to assess the immediate effects following a single session of stimulation (18, 21). The intervention dosage in the other trials varied, ranging from 15 to 66 minutes per session, with 1 to 2 sessions conducted per day, 1 to 7 times per week, over a period of 2 to 20 weeks (22–27).

### Effects of Lo-TENS on individuals with EH

#### Changes over time within group

With the exception of one trial investigating the immediate impact (18), all other reviewed trials reported a notable decrease in both systolic blood pressure (SBP) and diastolic blood pressure (DBP) in the Lo-TENS group. The reductions observed post-intervention ranged from 4.7 to 8.5 mmHg for SBP and 5.8 mmHg for DBP, with follow-up reductions of 6.7 to 7.1 mmHg for SBP and 2.8 to 4.9 mmHg for DBP. However, the decrease in DBP was not significant at post-intervention in one trial (22), nor was it significant at the 24-week follow-up in another (23). It is important to note that these significant reductions were all determined through office measurements. In contrast, when ambulatory blood pressure monitoring (ABPM) was used, none of the changes in blood pressure at post-intervention or at follow-up reached statistical significance. Additionally, one trial demonstrated that a single session of Lo-TENS significantly reduced the low-frequency (0.04 to 0.15 Hz) components (LF) and increased the normalized units of high-frequency (0.15 to 0.40 Hz) components (HF) of heart rate variability (HRV). This same trial also reported significant decrease in the LF/HF ratio after stimulation, indicating a potential shift towards increased parasympathetic activity (18) (as detailed in **Table 2**).

**Table 2:** TENS intervention: Effects on hypertension (N=4).

Outcomes	Within-group comparison		Between-group comparison	
	Experimental group	Control group	Comparison with no intervention	Comparison with other intervention
**Systolic blood pressure (mmHg) (N=4)**	**Immediate effect:** ↓^*^ (21), (NS) (18) **Post-intervention:** Office measurement: ↓^*^ (21–23); ABPM: (NS) (22); **4-week follow-up:** Office measurement: ↓^*^ (22); ABPM: (NS) (22) **12-week follow-up:** ↓^*^ (23) **24-week follow-up:** ↓^*^ (23)	**Immediate effect:** Hi-TENS group, Sham control group: (NS) (18, 21) **Post-intervention:** Office measurement: Felodipin group: ↓^*^ (22); Sham control group: (NS) (21, 23); ABPM: Felodipin group: 24-h ↓^*^, daytime ↓^*^ (22) **4-week follow-up:** Felodipin group: ↓^*^ (22); ABPM: (NS) (22) **12-week follow-up:** Sham control group: ↓^*^ (23) **24-week follow-up:** Sham control group: (NS) (23)	**Immediate effect:** ↓^*^ (21), (NS) (18) **Post-intervention:** ↓^*^ (21, 23) **12-week follow-up:** ↓^*^ (23) **24-week follow-up:** (NS) (23)	**Immediate effect:** Hi-TENS group: (NS) (18) **Post-intervention:** Felodipin group: ↑^*^ (22) **4-week follow-up:** Felodipin group: (NS) (22)
**Diastolic blood pressures (mmHg) (N=4)**	**Immediate effect:** ↓^*^ (21), (NS) (18) **Post-intervention:** Office measurement: (NS) (22); ↓^*^ (21, 23); ABPM: (NS) (22); **4-week follow-up:** Office measurement: ↓^*^ (22); ABPM: (NS) (22) **12-week follow-up:** ↓^*^ (23) **24-week follow-up:** (NS) (23)	**Immediate effect:** Hi-TENS group: ↑^*^ (18); Sham control group: (NS) (21) **Post-intervention:** Office measurement: Felodipin group: ↓^*^ (22); Sham control group: (NS) (21, 23); ABPM: (NS) (22); **4-week follow-up:** Office measurement: Felodipin group: ↓^*^ (22); ABPM: Felodipin group: (NS) (22) **12-week follow-up:** (NS) (23) **24-week follow-up:** (NS) (23)	**Immediate effect:** ↓^*^ (21), (NS) (18) **Post-intervention:** (NS) (21, 23) **12-week follow-up:** (NS) (23) **24-week follow-up:** (NS) (23)	**Immediate effect:** Hi-TENS group: (NS) (18) **Post-intervention:** Felodipin group: ↑^*^ (22) **4-week follow-up:** Felodipin group: (NS) (22)
**Mean blood pressure (mmHg) (N=1)**	**Immediate effect:** ↓^*^ (21); **Post-intervention:** ↓^*^ (21)		**Immediate effect:** ↓^*^ (21); **Post-intervention:** ↓^*^ (21)	
**Others (N=3)**	**Immediate effect:** **Heart Rate Variability:** LF (%): ↓^*^, LF (n.u.): (NS), HF (%): (NS), HF (n.u.): ↑^*^, LF/HF index: ↓^*^ (18) **Post-intervention:** **Mean Heart Rate:** (NS) (22)	**Immediate effect:** **Heart Rate Variability:** LF (%), LF (n.u.), HF (n.u.), HF (%), LF/HF index: Hi-TENS group, Sham control group: (NS) (18) **Post-intervention:** **Mean Heart Rate:** Felodipin group: (NS) (22)	**Immediate effect:** **Heart Rate Variability:** LF (%), LF (n.u.), HF (n.u.), HF (%), LF/HF index: (NS) (18)	**Immediate effect:** **Heart Rate Variability:** LF (%), LF (n.u.), HF (n.u.), HF (%), LF/HF index: Hi-TENS group: (NS) (18) **Post-intervention:** **Mean Heart Rate:** Felodipin group: (NS) (22)

*: Significant difference; ↑: increase; ↓: decrease; N: number of included trials; NS: not significant.

AMBP, ambulatory monitoring of blood pressure; HF (%), high-frequency component; HF (n.u.), normalized unit of high-frequency; Hi-TENS, high frequency transcutaneous electrical nerve stimulation; LF (n.u.), normalized unit of low-frequency; LF(%), low-frequency component; Hi-TENS, high frequency transcutaneous electrical nerve stimulation.

#### Between-group comparison

In the comparison between the Lo-TENS and placebo groups, the Lo-TENS group experienced a more pronounced decrease in SBP and mean BP following a single session of stimulation, with these reductions being evident at post-intervention and subsequent follow-ups (21, 23). However, when examining DBP, no significant between-group differences were observed at either post-intervention or follow-up, except for a notable greater reduction in the Lo-TENS group immediately after stimulation in one instance (21).

Furthermore, when the Lo-TENS group was compared with a group treated with the antihypertensive medication felodipine, the Lo-TENS group exhibited a significantly higher SBP (by 5.3 mmHg) and DBP (by 4.8 mmHg) at the completion of the intervention (22). As for heart rate variability (HRV) measures, no significant between-group differences were noted when the Lo-TENS group was compared to either the Hi-TENS or placebo control groups (18) (refer to **Table 2** for details).

### Effects of Lo-TENS on individuals with T2DM

#### Changes over time within group

Over the course of the study, two trials documented notable improvements in various glycemic (glycosylated hemoglobin [HbA1c], fasting plasma glucose [FPG], 2-hour postprandial glucose [2hPG], mean amplitude of glycemic excursions [MAGE], fructosamine [FISN]) and lipid (total cholesterol [TC], triglycerides [TG]) parameters, as well as in levels of certain biomarkers (C-reactive protein [CRP], tumor necrosis factor-alpha [TNF-α], adiponectin, fibroblast growth factor 21 [FGF-21]). However, some of these improvements, specifically in TNF-α and adiponectin levels, did not reach statistical significance (24, 25) (as detailed in **Table 3**).

**Table 3:** TENS intervention: Effects on diabetes (N=2).

Outcomes	Within-group comparison		Between-group comparison	
	Experimental group	Control group	Comparison with no intervention	Comparison with other intervention
**Glycemia profile**	**Post-intervention:** **HbAlc (%):** ↓^*^ (24, 25); **FPG, MAGE:** ↓^*^ (25); **2hPG, FISN:** ↓^*^ (24) **Follow-up:** **HbAlc (%), 2hPG, FISN:** ↓^*^ (24)	**Post-intervention:** **HbAlc (%):** Sham control group: ↓^*^ (25), (NS) (24); Aerobic exercise group: ↓^*^ (24); **2hPG, FISN:** Sham control group: (NS) (24); Aerobic exercise group: ↓^*^ (24); **FPG, MAGE:** Sham control group: (NS) (25) **Follow-up:** **HbAlc (%), 2hPG, FISN:** Sham control group: (NS); Aerobic exercise group: ↓^*^ (24)	**Post-intervention:** **HbAlc (%):** (NS) (25), ↓^*^ (24); **FPG:** (NS) (25); **2hPG, MAGE, FISN:** ↓^*^ (24). **Follow-up:** **HbAlc (%), 2hPG, FISN:** (NS) (24)	**Post-intervention:** **HbAlc (%), 2hPG, FISN:** Aerobic exercise group: (NS) (24) **Follow-up:** **HbAlc (%), 2hPG, FISN:** Aerobic exercise group: (NS) (24)
**Lipid profile**	**Post-intervention:** **TC, TG:** ↓^*^ (24) **Follow-up:** **TC, TG:** ↓^*^ (24)	**Post-intervention:** **TC, TG:** Sham control group: (NS); Aerobic exercise group: ↓^*^ (24) **Follow-up:** **TC, TG:** Sham control group: (NS); Aerobic exercise group: ↓^*^ (24)	**Post-intervention:** **TC, TG:** ↓^*^ (24) **Follow-up:** **TC, TG:** (NS) (24)	**Post-intervention:** **TC, TG:** Aerobic exercise group: (NS) (24) **Follow-up:** **TC, TG:** Aerobic exercise group: (NS) (24)
**Exploratory biomarkers**	**Post-intervention:** **CRP, FGF-21:** ↓^*^; **TNF-α, Adiponectin:** (NS) (25)	**Post-intervention:** **FGF-21, CRP, TNF-α, Adiponectin:** Sham control group: (NS) (25)	**Post-intervention:** **CRP, FGF-21:** ↓^*^; **TNF-α, Adiponectin:** (NS) (25)	

*: Significant difference; ↓: decrease; N: number of included trials; NS: not significant.

2hPG: 2 h postprandial glucose; HbA1c (%): glycosylated hemoglobin; FPG: fasting plasma glucose; FISN: fasting serum insulin; TC: total cholesterol; TG: triglyceride; CRP: C-reactive protein; TNF-α: tumor necrosis factor-α; FGF-21: fbroblast growth factor-21; MAGE: mean amplitude of glycemic excursion.

#### Between-group comparison

In comparisons with the placebo control group, a consistent pattern of improvement was observed in the Lo-TENS group, with the exception of one trial where two glycemic measures—HbA1c and FPG—did not exhibit significant between-group differences at post-intervention (25). When the Lo-TENS group was compared with an aerobic exercise group, no significant between-group differences were found for the aforementioned outcomes at either post-intervention or follow-up measurements (24). However, the Lo-TENS group did show significantly lower levels of CRP and FGF-21 compared to the placebo control group (25) (refer to **Table 3** for detailed results).

### Effects of Lo-TENS on individuals with obesity

#### Changes over time within group

Within-group analysis indicated that a significant reduction was observed only in waist circumference at post-intervention (27). No other significant improvements were noted in anthropometric measures (weight, body mass index [BMI], adipose tissue area), lipid profiles (TC, TG, high-density lipoprotein cholesterol [HDL-C], free fatty acids [FFA]), glycemic indicators (HbA1c, glucose, insulin, homeostatic model assessment of insulin resistance [HOMA-IR]), or exploratory biomarkers (high-sensitivity C-reactive protein [HsCRP], ghrelin, growth hormone [GH], insulin-like growth factor 1 [IGF-1]) (26, 27) (as detailed in **Table 4**).

**Table 4:** TENS intervention: Effects on obesity (N=2).

Outcomes	Within-group comparison		Between-group comparison	
	Experimental group	Control group	Comparison with no intervention	Comparison with other intervention
**Anthropometric measures (N=2)**	**Post-intervention:** **Weight, BMI:** (NS) (26, 27); **Waist circumference:** ↓^*^ (27); **Adipose tissue area** (VAF, SAF, TAF): (NS) (27) **1 month follow-up:** **Weight, BMI:** (NS) (26)	**Post-intervention:** **Weight, BMI:** EMS group: ↓^*^ (27); PENS group, Diet group: (NS) (26); **Waist circumference:** EMS group ↓^*^ (27) **Adipose tissue area** (VAF, SAF, TAF): (NS) (27) **1 month follow-up:** **Weight, BMI:** PENS group, Diet group: (NS), PENS+Diet Group: ↓^*^ (26)	**Post-intervention:** **Weight, BMI:** (NS) (26) **1 month follow-up:** **Weight, BMI:** (NS) (26)	**Post-intervention:** **Weight, BMI:** EMS group: (NS) (27); PENS group: (NS), PENS+diet group: ↑^*^ (26); **Adipose tissue area** (VAF, SAF, TAF): (NS) (27); **Waist circumference:** EMS group: ↑^*^ (27) **1 month follow-up:** **Weight, BMI:** PENS group: (NS), PENS+Diet group: ↑^*^ (26)
**Lipid profile (N=2)**	**Post-intervention:** **TC, TG, HDL-C:** (NS) (26, 27); **FFA:** (NS) (27) **1 month follow-up:** **HDL-C:** (NS) (26)	**Post-intervention:** **TC, HDL-C:** EMS group, PENS group, Diet group, PENS+Diet group: (NS) (26, 27); **TG:** EMS group, PENS group, Diet group: (NS) (26, 27); PENS + Diet group: ↓^*^ (26) **FFA:** EMS group: ↑^*^ (27) **1 month follow-up:** **TC, HDL-C:** PENS group, Diet group, PENS+Diet group: (NS) (26) **TG:** PENS group, Diet group: (NS), PENS + Diet group: ↓^*^ (26)	**Post-intervention:** **TC, TG, HDL-C:** (NS) (26) **1 month follow-up:** **TC, TG, HDL-C:** (NS) (26)	**Post-intervention:** **TC, HDL-C:** EMS group, PENS group, PENS + Diet group: (NS) (26, 27) **TG:** EMS group, PENS group: (NS) (26, 27); PENS+Diet group: ↑^*^ (26) **FFA:** ↑^*^ (27) **1 month follow-up:** **TC, HDL-C:** PENS group, PENS + Diet group: (NS) (26) **TG:** PENS group: (NS), PENS+Diet group: ↑^*^ (26)
**Glycemia profile (N=2)**	**Post-intervention:** **HbAlc, Glucose, Insulin, HOMA-IR:** (NS) (26, 27); **1 month follow-up:** **HbAlc, Glucose, Insulin, HOMA-IR:** (NS) (26)	**Post-intervention:** **HbAlc, Insulin, HOMA-IR:** EMS group, PENS group, Diet Group, PENS+Diet group: (NS) (26, 27); **Glucose:** EMS group, PENS group, Diet Group: (NS) (26, 27); PENS+Diet group: ↓^*^ (26) **1 month follow-up:** **Glucose, Insulin:** PENS group, Diet group, PENS+Diet group: (NS); **HbAlc, HOMA-IR:** PENS group, Diet group: (NS); PENS+Diet group: ↓^*^ (26)	**Post-intervention:** **HbAlc, Glucose, Insulin, HOMA-IR:** (NS) (26) **1 month follow-up:** **HbAlc, Glucose, Insulin, HOMA-IR:** (NS) (26)	**Post-intervention:** **HbAlc, Insulin, HOMA-IR:** EMS group, PENS group, PENS+Diet group: (NS) (26, 27); **Glucose:** EMS group, PENS group: (NS) (26, 27); PENS+Diet group: ↑^*^ (26) **1 month follow-up:** **Insulin:** PENS group, PENS+Diet group (NS); **HbAlc, Glucose, HOMA-IR:** PENS group (NS), PENS+Diet group: ↑^*^ (26)
**Exploratory biomarkers (N=2)**	**Post-intervention:** **HsCRP:** (NS) (27); **Ghrelin, GH, IGF-1:** (NS) (26) **1 month follow-up:** **Ghrelin, GH, IGF-1:** (NS) (26)	**Post-intervention:** **HsCRP:** EMS group: (NS) (27) **Ghrelin, GH, IGF-1:** Diet Group: (NS), PENS+Diet group, PENS group: ↓^*^ (26) **1 month follow-up:** **Ghrelin, GH, IGF-1:** Diet Group: (NS), PENS+Diet group, PENS group: ↓^*^ (26)	**Post-intervention:** **Ghrelin, GH, IGF-1:** (NS) (26) **1 month follow-up:** **Ghrelin, GH, IGF-1:** (NS) (26)	**Post-intervention:** **HsCRP:** EMS group: (NS) (27) **Ghrelin, GH, IGF-1:** PENS+Diet group, PENS group: ↑^*^ (26) **1 month follow-up:** **Ghrelin, GH, IGF-1:** PENS+Diet group, PENS group: ↑^*^ (26)

*: Significant difference; ↑: increase; ↓: decrease; N, number of included trials; NS, not significant.

BMI, body mass index; EMS, electrical muscle simulation; FFA, free fatty acid; GH, growth hormone; HbA1c, glycosylated hemoglobin; HDL-C, high density lipoprotein cholesterol; HOMA-IR, homeostasis model assessment insulin resistance index; hsCRP, high sensitivity C-reactive protein; IGF-1, insulin-like growth factor 1; PENS, percutaneous electrical nerve stimulation; SAF, subcutaneous abdominal fat area; TAF, total abdominal fat area; TC, total cholesterol; TG, triglyceride; VAF: visceral abdominal fat area.

#### Between-group comparison

Comparative analysis between groups revealed no significant changes when the Lo-TENS group was compared to the placebo control group. However, when compared to other intervention groups, the results were inconsistent. Overall, the Lo-TENS group demonstrated comparable efficacy to the physical exercise and percutaneous electrical nerve stimulation (PENS) group and the electrical muscle stimulation (EMS) group (26, 27). In contrast, the Lo-TENS group showed less effectiveness than the PENS combined with dietary intervention group in terms of changes in anthropometric, lipid, glycemic, and exploratory biomarker outcomes (summarized in **Table 4**).

## Discussion

### Findings of this review

This review encompassed eight RCTs that assessed the impact of Lo-TENS on three distinct populations, comparing its effects against no intervention or other forms of intervention. Relative to pre-intervention measurements, Lo-TENS appeared to favorably influence blood pressure (BP) in individuals with EH and metabolic parameters in those with T2DM. However, its effectiveness in managing obesity remains uncertain. A positive trend was also observed in comparisons with the no-intervention group, suggesting that Lo-TENS may be beneficial for individuals with EH and T2DM. When Lo-TENS was juxtaposed with other interventions, three trials indicated less favorable outcomes. Nonetheless, definitive conclusions cannot be drawn due to the limited number of trials included in this review.

Previous original studies have indicated that Lo-TENS may lead to reductions in SBP and DBP, as evidenced by pre-post intervention comparisons (28, 29). These findings align with the majority of the trials reviewed here, which demonstrate significant within-group improvements in various health outcomes following Lo-TENS intervention. Moreover, this effectiveness is further corroborated by the between-group analysis showing that the Lo-TENS group experienced greater improvements in most outcomes compared to the no-intervention group.

In exploring whether Lo-TENS could serve as an adjunctive treatment for obesity and associated conditions, three key criteria must be met. Firstly, it is essential to establish whether Lo-TENS effectively improves outcomes related to obesity and its associated conditions. The within-group and between-group analysis provide support for this effectiveness. Secondly, it is crucial to ensure that Lo-TENS is safe to administer without causing adverse events. The reviewed articles affirm this safety profile, with no adverse events reported across the trials. Lastly, Lo-TENS should demonstrate a level of efficacy at least comparable to other interventions.

In the comparison with the felodipine group, the between-group analysis indicated that the effect of Lo-TENS on BP in EH population was less pronounced, with a greater reduction in SBP and DBP observed in the felodipine group by 5.3 and 4.8 mmHg, respectively (22). However, the interpretation of these results must be approached with caution due to the small sample size of 32 participants and the crossover design of the study. Additionally, no significant outcomes were measured by ambulatory monitoring of blood pressure (AMBP), and these findings are based on data from a single trial.

As for the impact of Lo-TENS on biochemical and lipid profiles in the T2DM population, the between-group analysis comparing Lo-TENS to aerobic exercise showed no significant difference in effects (24). This is clinically significant, as numerous guidelines recommend exercise as the primary treatment for T2DM (30, 31). Exercise is known to enhance insulin sensitivity and improve both blood glucose levels and overall physical fitness (32). However, the effectiveness of aerobic exercise varies, and a certain threshold of duration and intensity is necessary to achieve benefits, with research suggesting a minimum of 30 minutes of exercise several times a week (33). For some older patients, particularly those with arthritis, heart disease, or limited mobility, maintaining a regular exercise regimen can be challenging. This is especially true for T2DM patients, who are often older and may contend with comorbidities such as heart disease, cerebral vascular disease, or bone and joint disorders (34), making long-term adherence to an exercise program difficult. Therefore, alternative non-pharmacological treatments, including electrical stimulation, may be a valuable addition to the treatment arsenal for these patients (35). Given that Lo-TENS has been shown to have effects comparable to aerobic exercise, it may serve as a valuable supplementary strategy in the management of T2DM. Moreover, one trial indicated that the Lo-TENS group experienced a lower MAGE, suggesting better control of glycemic variability (25). This is particularly important in T2DM management, where avoiding both hyperglycemia and hypoglycemia is crucial.

Diabetes management typically involves pharmacological interventions, including medications such as metformin and acarbose, as well as insulin therapy. While these treatments have been instrumental in saving numerous lives and are generally effective, they are not universally optimal for every patient. For instance, metformin has been associated with the risk of lactic acidosis in rare cases (36), and insulin therapy can lead to weight gain (37) and other complications. Recognizing the limitations of single-treatment approaches, a combined therapeutic strategy may offer a more tailored and effective solution for diabetes management. Lo-TENS as an adjunctive therapy, could potentially complement drug treatments, allowing for a reduction in drug dosages and, consequently, a mitigation of associated risks. For example, integrating Lo-TENS with metformin therapy might enable a lower metformin dosage, thereby reducing the likelihood of lactic acidosis.

In the context of obesity, although the between-group analysis of other interventions revealed that the Lo-TENS group did not fare as well as the group receiving PENS plus diet intervention (in terms of weight, BMI, TG, HbA1c, glucose, and HOMA-IR) or the EMS group (in terms of waist circumference and FFA), the other comparisons did not show significant differences (26, 27). Considering that PENS is an invasive procedure, patient tolerance and compliance could limit its application. Furthermore, the greater reduction in waist circumference observed in the EMS group was reported in one trial only, thus, this result should be approached with caution.

Among the trials included in this review, only one assessed the impact of Lo-TENS on the sympathetic and parasympathetic nervous systems, and this study involved a single session of intervention (18). Consequently, the immediate effects observed do not permit definitive conclusions. While some studies have demonstrated the efficacy of Lo-TENS in reducing sympathetic activity in healthy individuals (17, 29), its effectiveness in populations with obesity and associated conditions remains unexplored. Prior reviews have suggested that inhibiting the overactive sympathetic nervous system could be advantageous for those with obesity and associated conditions, such as EH, T2DM, and dyslipidemia (2, 6, 38). In this review, none of the eight trials examined the potential pathway from Lo-TENS to sympathetic activity and then to obesity and associated conditions. Further research is needed to evaluate the effectiveness of Lo-TENS and to uncover the mechanisms by which it may exert its effects.

Three of the reviewed trials investigated changes in biomarkers, which could provide insights into the possible pathways through which Lo-TENS operates (25–27). However, none of the trials analyzed the link between changes in biomarkers and changes in clinical outcomes, making it impossible to determine the connection between these changes and improved treatment results.

Sub-group analysis revealed that among individuals with T2DM, those who were female, had a baseline BMI of 26.9 kg/m^2^ or higher, or had a disease onset of 9 years or more tended to experience better treatment outcomes (25). These findings suggest that participants’ gender, baseline BMI, and duration of disease onset might be significant factors in determining the success of treatment interventions. Further investigation into how these demographic and clinical characteristics affect treatment outcomes is encouraged to enhance the precision and efficacy of therapeutic strategies in the future.

### Limitations of trials reviewed

In this review, all eight included trials were rated as being at high risk of bias, which weakens the strength of the evidence provided. Among these, four trials reported on the persistence of intervention effects (22–24, 26), whereas the remaining four did not mention such effects (18, 21, 25, 27). Notably, only one trial implemented a single-session stimulation protocol (18). These limitations hinder a comprehensive understanding of the true efficacy of Lo-TENS for obesity and associated conditions.

To better assess the potential of Lo-TENS in treating obesity and associated conditions, there is a need for more rigorously designed studies with longer intervention periods and clear documentation of the retention of intervention effects. Such RCTs would provide more robust evidence to determine the actual effectiveness of Lo-TENS in this context.

### Limitations of this review

The search for relevant articles was limited to English-language databases, which may have overlooked potentially pertinent publications in other languages. This limitation could affect the breadth and applicability of the findings. Additionally, the variability in populations and outcomes across the reviewed trials precludes the conduction of a meta-analysis, which would otherwise synthesize the data and provide a more unified understanding of the results.

### Clinical and research implications

The insights gained from this review offer valuable guidance for healthcare professionals. Firstly, the absence of adverse events in the included trials suggests that integrating Lo-TENS into the standard care for obesity and associated conditions is a feasible option. Secondly, given its cost-effectiveness and simplicity, Lo-TENS warrants further consideration from both clinicians and researchers, especially as it has been shown to improve various outcomes across different populations. Thirdly, the selection of stimulation parameters across the reviewed trials provides a basis for developing new intervention protocols, which could be tailored to specific patient needs. The gaps in knowledge highlighted by this review point towards areas that future research should address. Subsequent studies should thoroughly evaluate the effectiveness of Lo-TENS in treating obesity and a range of associated conditions, utilizing more rigorous study designs. Additionally, there is a need to clarify the underlying mechanisms of action of Lo-TENS. Comparative studies on the efficacy of different stimulation parameters, such as waveform, frequency, stimulation sites, and duration, are essential to formulate the most effective intervention strategies. Lastly, identifying the factors that contribute to successful treatment outcomes with Lo-TENS is an important area for investigation, as it could help to enhance patient selection and tailor interventions for optimal results.

## Conclusions

While Lo-TENS did not consistently outperform other interventions or showed only marginal improvements, it did generally elicit greater benefits compared to the majority of placebo controls. This suggests that Lo-TENS might be a valuable supplementary intervention for managing obesity and its associated conditions. However, due to the small number of trials reviewed, the high risk of bias inherent in these studies, and the limited evidence available, it is premature to draw definitive conclusions. The current findings should be approached with circumspection. There is an urgent need for additional high-quality research to further explore and confirm the effectiveness of Lo-TENS in the context of obesity and related disorders.

## Data Availability

The original contributions presented in the study are included in the article/**Supplementary Material**. Further inquiries can be directed to the corresponding author/s.
