# Supplementary material for: Adjunctive benefits of low-frequency transcutaneous electrical nerve stimulation for obesity frequent chronic conditions: a systematic review
Source: Front Endocrinol (Lausanne). 2024 Aug 9;15:1424771. doi: 10.3389/fendo.2024.1424771 (PMC11341397; doi:10.3389/fendo.2024.1424771)
Supplement: Supplementary file 1 [file DataSheet_1.docx]

Appendix 1. Search strategy

#1 essential hypertension

#2 hypertension

#3 high blood pressure

#4 hypercholesterolemia

#5 hyperlipidemia

#6 dyslipidemia

#7 diabetes

#8 diabetes mellitus

#9 type 2 diabetes mellitus

#10 insulin resistance

#11 obesity

#12 overweight

#13 metabolic syndrome

#14 metabolic diseases

#15 #1 OR #2 OR #3 OR #4 OR #5 OR #6 OR #7 OR #8 OR #9 OR #10 OR #11 OR #12 OR #13 OR #14

#16 transcutaneous electrical nerve stimulation

#17 TENS

#18 #16 OR #17

#19 #15 AND #18 Limit to Human English
